# Supplementary material for: Interplay between the Chd4/NuRD Complex and the Transcription Factor Znf219 Controls Cardiac Cell Identity
Source: Int J Mol Sci. 2022 Aug 24;23(17):9565. doi: 10.3390/ijms23179565 (PMC9455175; doi:10.3390/ijms23179565)
Supplement: Supplementary file 1 [file ijms-23-09565-s001.zip › ijms-1863636-supplementary/Suppl/Figures S1-S7.pdf]

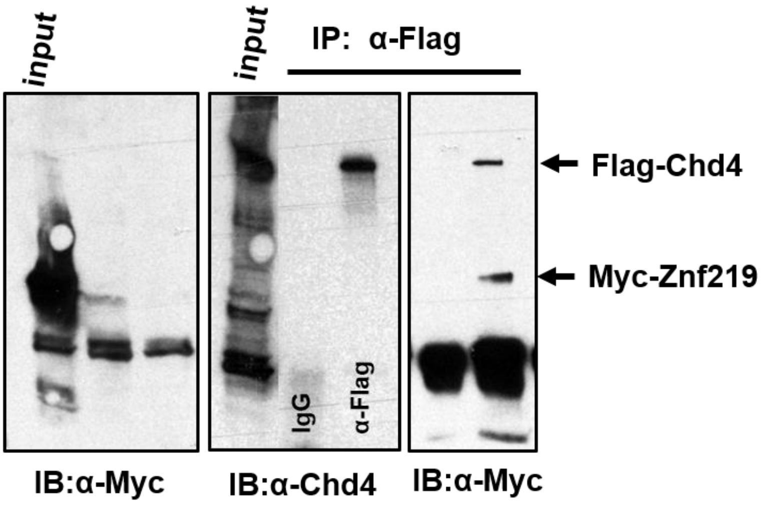

Figure S1. Chd4 and Znf219 interact in HEK293 cells. Western blot showing Hek293 extracts co-transfected with Flag-Chd4 and Myc-Znf219 (input), and IP with anti-Flag showing *in vitro* interaction of both proteins.

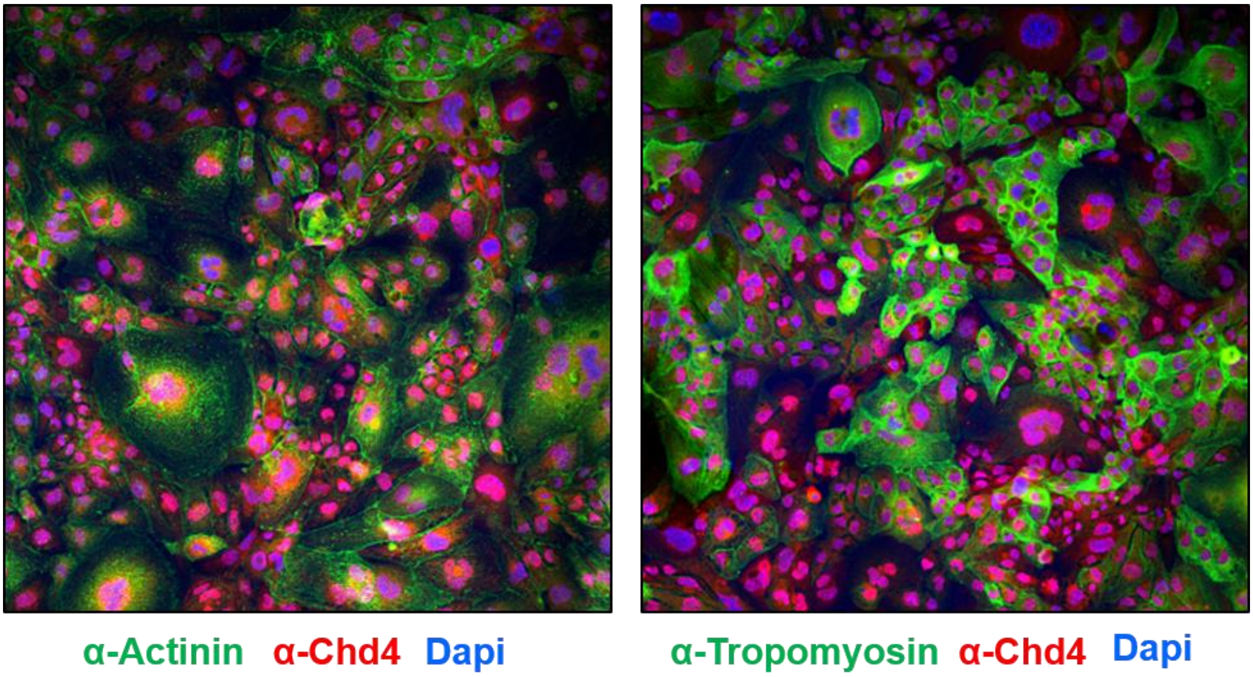

Figure S2. HL-1 cell line characterization. IF of HL-1 cells showing expression of the cardiac markers α-actinin (left) and tropomyosin (right, 84% +/- 4%) in green. Chd4 immunostaining is showed in red

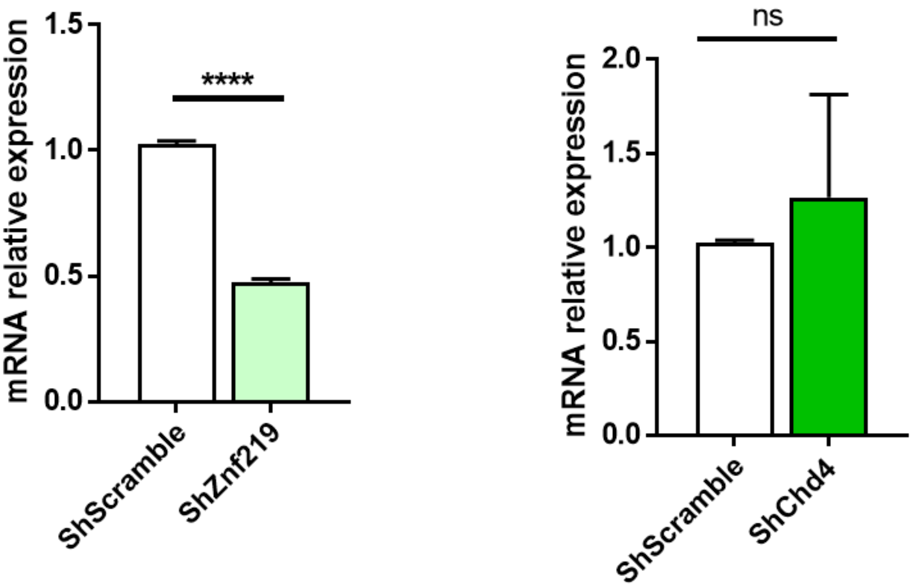

Figure S3. ***Znf219* KD *in vitro*.** qPCRs of HL-1 cells infected with shRNA for *Znf219* and *Chd4* of samples showed in Figure 2e. Left chart shows KD of *Znf219* with its corresponding shRNA. Right chart shows no *Znf219* KD when cells were infected with shRNA for *Chd4*. Cells infected with scrambled shRNA were used as control.

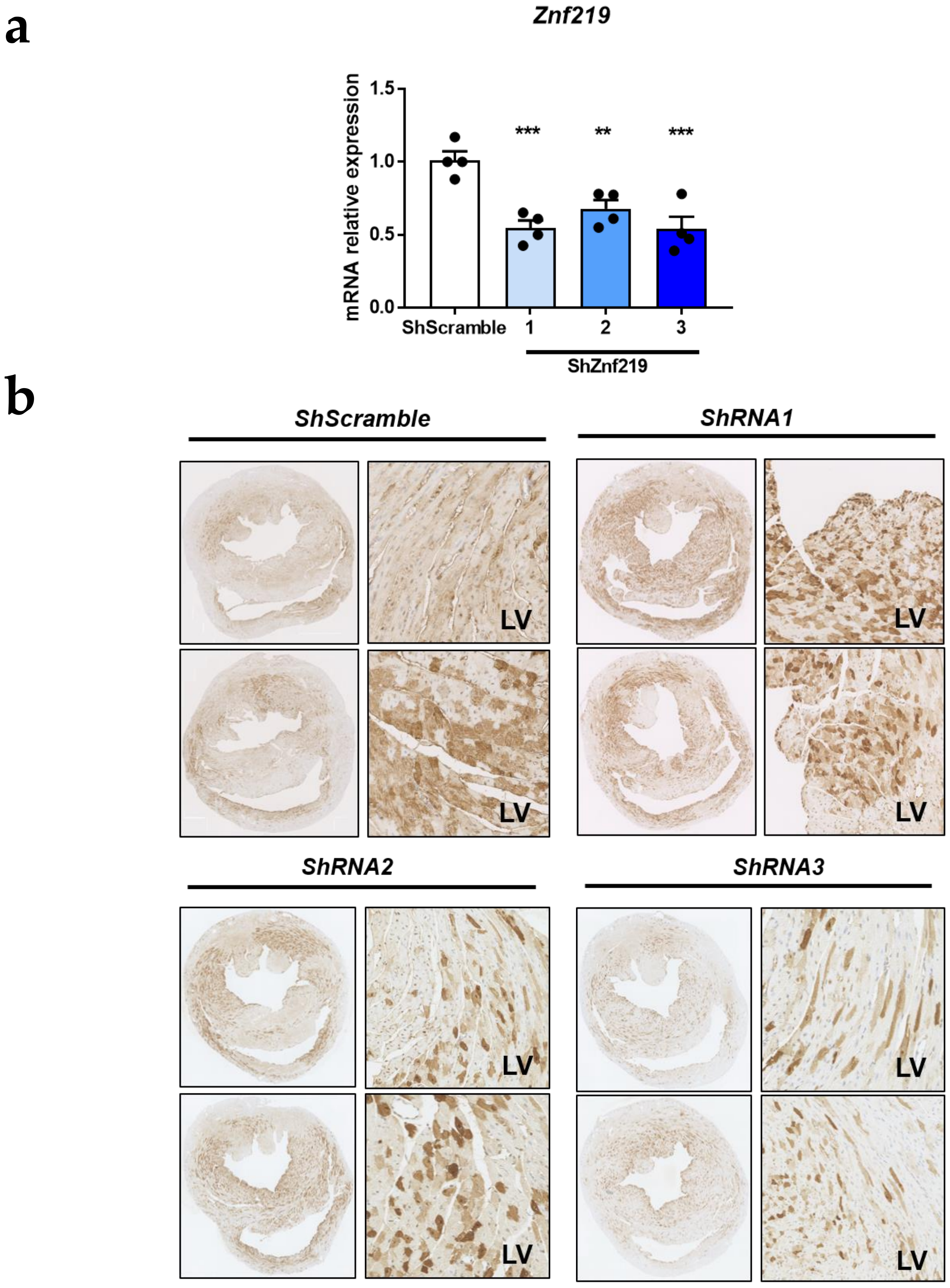

Figure S4. ***Znf219*** **KD** ***in vivo*****.** (**a**) WT CD1 PD1-2 pups were injected with 2x10^{11} viral particles/pup with AAV9-shRNA-*ShScramble* or AAV9-shRNA1-,2- or 3-*Znf219*. One month later, RNA from the hearts of these mice were tested for *Znf219* KD by qPCR. (**b**) Levels of infection were tested by IHC with α-GFP, showing good, but mosaic cardiomyocyte infection with all three shRNAs tested.

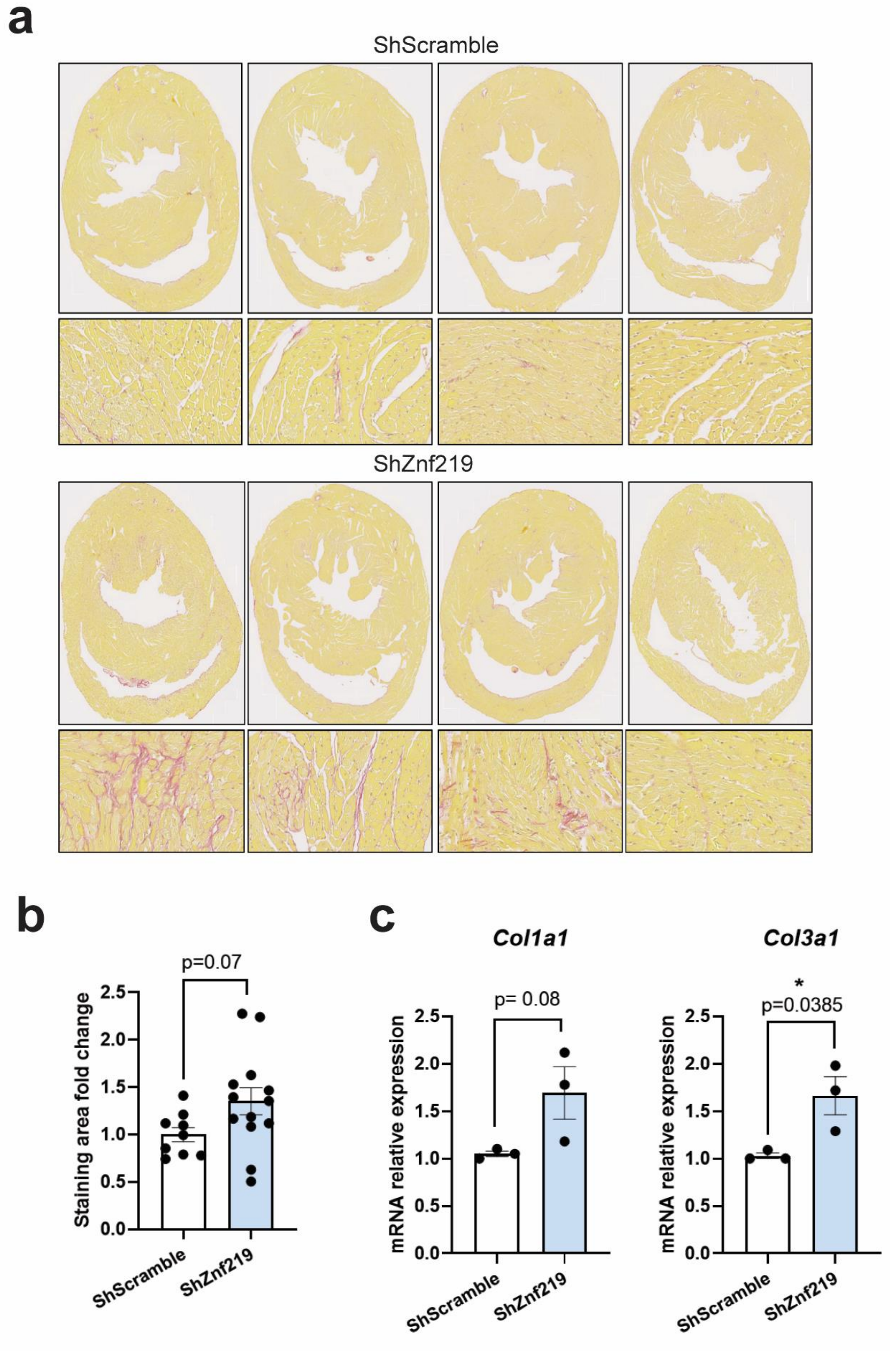

Figure S5. ***Znf219*** **KD hearts tend to increase fibrosis and collagen transcription.** (**a**) Sirius Red staining of heart transversal sections of 4 representative 8 week old infected mice with AAV9-*shScramble* (upper), and 4 infected with AAV9-sh-RNA *Znf219*. (**b**) Quantification of fibrotic area in 9 sh-scramble infected hearts (white bar), and 13 sh*Znf219* infected hearts (blue bar). (**c**) *Collagen type I alpha 1 (Col1a1)* and type III alpha 1 (Col*3a1)* mRNA levels in shScramble or sh *Znf219* infected hearts (n=3).

a

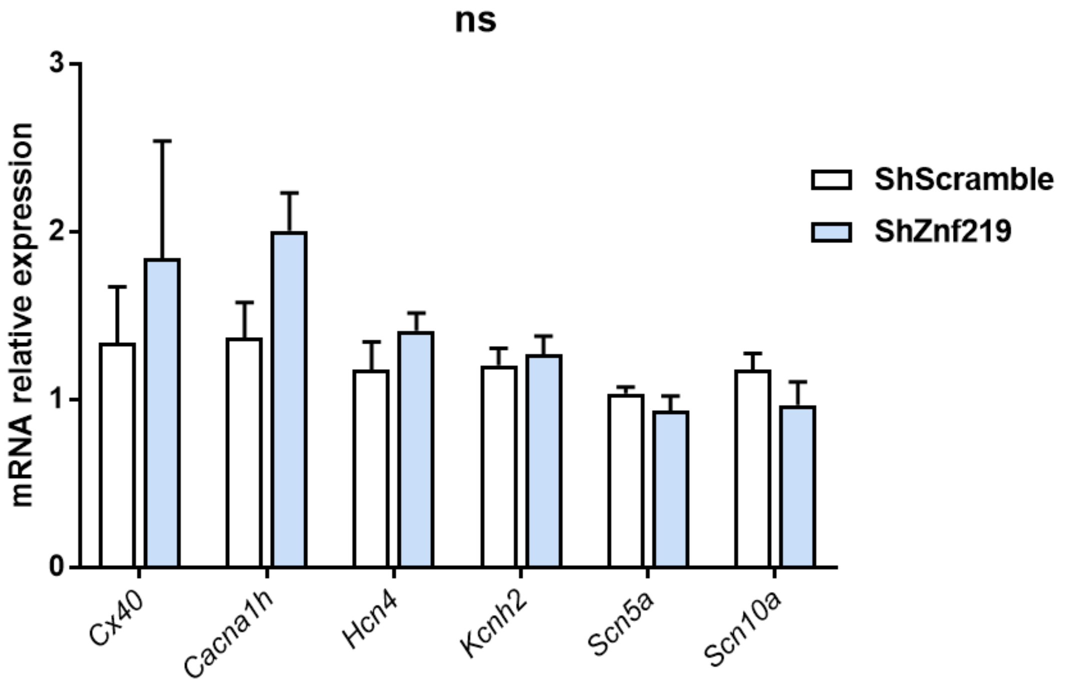

b

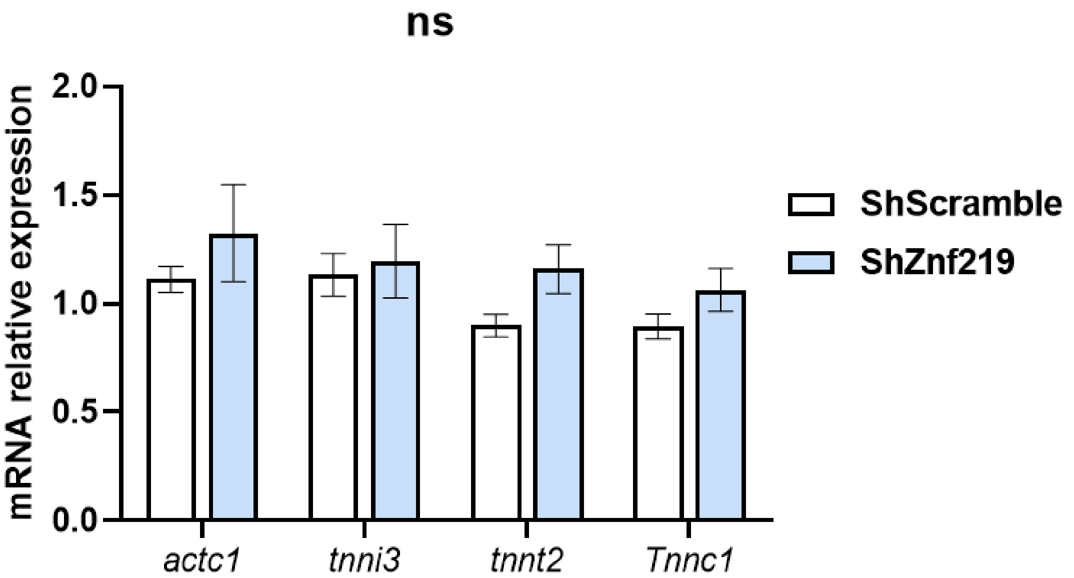

Figure S6. Znf219 does not regulate ion channels transcription ***in vivo or cardiac genes*****.** *Znf219* KD with Sh*Znf219* does not affect mRNA expression levels of some sodium, potassium or calcium channels (**a**), or sarcomeric cardiac genes showed by qPCR (**b**). ns = not significant

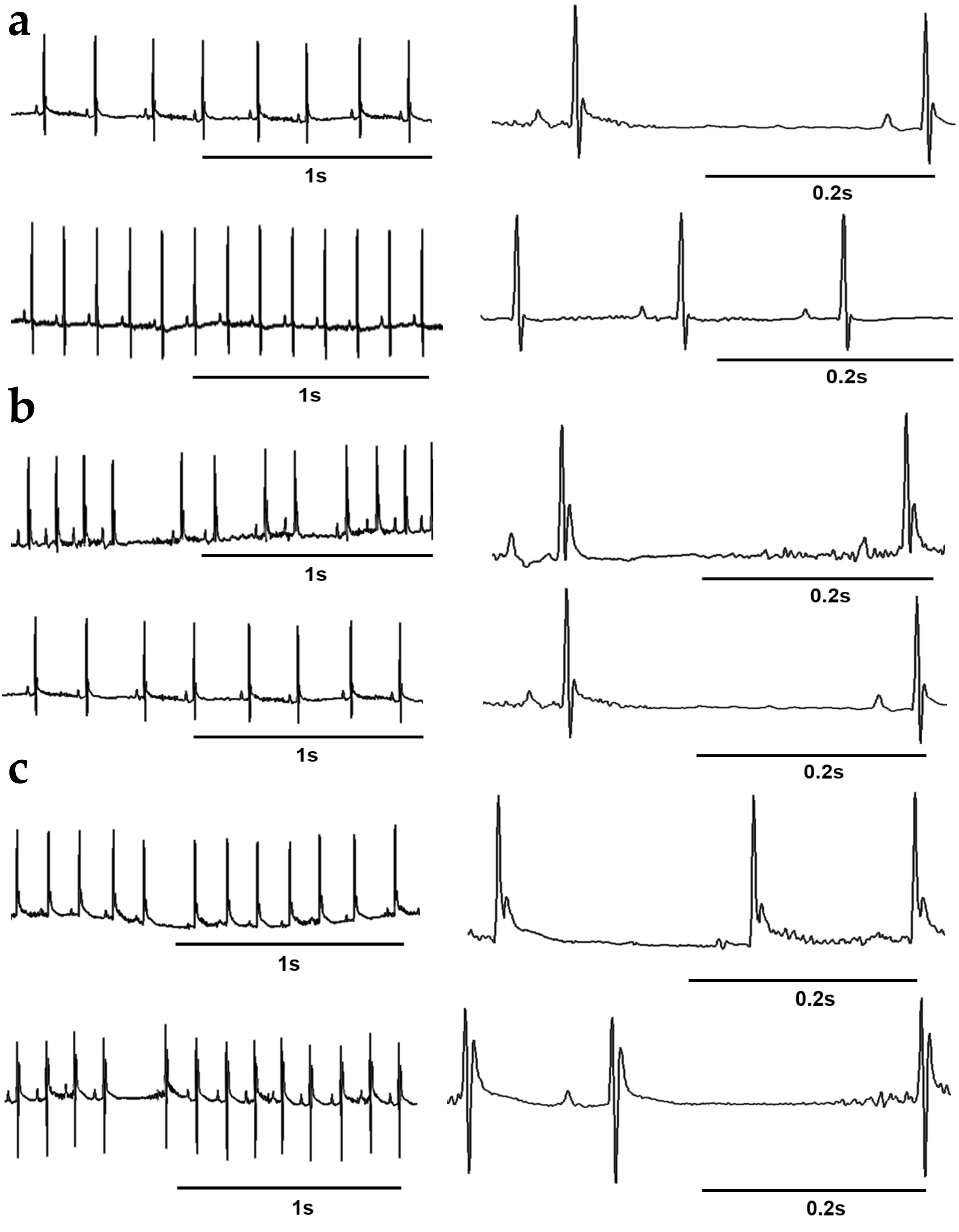

Figure S7. Representative ECGs. (a) ECGs of *ShScramble* 4 weeks old (upper) and 8 weeks-old infected animals (lower). **(b)** Representative arrhythmias of 4 week old animals infected with *ShZnf219* (n=2). **(c)** Representative arrhythmias of 8 weeks old *ShZnf219* infected animals (n=2).
